# Self-collection based HPV testing for cervical cancer screening among women living with HIV in Uganda: a descriptive analysis of knowledge, intentions to screen and factors associated with HPV positivity

**DOI:** 10.1186/s12905-016-0360-0

**Published:** 2017-01-13

**Authors:** Sheona M. Mitchell, Heather N. Pedersen, Evelyn Eng Stime, Musa Sekikubo, Erin Moses, David Mwesigwa, Christine Biryabarema, Jan Christilaw, Josaphat K. Byamugisha, Deborah M. Money, Gina S. Ogilvie

**Affiliations:** 1 University of British Columbia, Vancouver, BC Canada; 2 Makerere University, Kampala, Uganda; 3 Women’s Health Research Institute, Vancouver, BC Canada; 4 Kisenyi Health Centre, Kampala, Uganda; 5 BC Women’s Hospital and Health Centre, Box 42, Room H203G - 4500 Oak Street, Vancouver, BC V6H 3 N1 Canada

**Keywords:** Human papillomavirus, HIV, Cervical cancer, Self-collection, Screening

## Abstract

**Background:**

Women living with HIV (WHIV) are disproportionately impacted by cervical dysplasia and cancer. The burden is greatest in low-income countries where limited or no access to screening exists. The goal of this study was to describe knowledge and intentions of WHIV towards HPV self-collection for cervical cancer screening, and to report on factors related to HPV positivity among women who participated in testing.

**Methods:**

A validated survey was administered to 87 HIV positive women attending the Kisenyi Health Unit aged 30–69 years old, and data was abstracted from chart review. At a later date, self-collection based HPV testing was offered to all women. Specimens were tested for high risk HPV genotypes, and women were contacted with results and referred for care. Descriptive statistics, Chi Square and Fischer-exact statistical tests were performed.

**Results:**

The vast majority of WHIV (98.9%) women did not think it necessary to be screened for cervical cancer and the majority of women had never heard of HPV (96.4%). However, almost all WHIV found self-collection for cervical cancer screening to be acceptable. Of the 87 WHIV offered self-collection, 40 women agreed to provide a sample at the HIV clinic. Among women tested, 45% were oncogenic HPV positive, where HPV 16 or 18 positivity was 15% overall.

**Conclusions:**

In this group of WHIV engaged in HIV care, there was a high prevalence of oncogenic HPV, a large proportion of which were HPV genotypes 16 or 18, in addition to low knowledge of HPV and cervical cancer screening. Improved education and cervical cancer screening for WHIV are sorely needed; self-collection based screening has the potential to be integrated with routine HIV care in this setting.

## Background

Cervical cancer is a largely preventable and curable disease that affects over half a million women each year, with the greatest impact in low and middle income countries (LMIC) where access to screening, diagnosis, and treatment are extremely limited [1]. As a result, most people in these regions are diagnosed with cervical cancer at a late stage [2]. With this delayed diagnosis, more resources are required for treatment, which increases demands on already overloaded tertiary care centers. Uganda has one of the highest age-standardized incidence rates of cervical cancer globally (41.7/100 000 person-years) at almost six times the rate in high income countries, in addition to a high prevalence of human immunodeficiency virus (HIV) [3]. It is well established that oncogenic types of the human papillomavirus (HPV) are the main causative agents of cervical cancer [4]. Primary prevention with the HPV vaccine has not been rolled out in most LMIC including Uganda, and the risk for cervical cancer will remain for decades [5]. Characterized by suppressed immune function, women living with HIV (WHIV) are more likely to have persistent HPV infection and simultaneous infection with more than one strain of HPV putting them at higher risk for cervical dysplasia and invasive cervical cancer [6–8]. Cervical cancer is an AIDS-defining illness [9] that remains a threat in the era of combination antiretroviral treatment (cART) which have little to no effect on the natural history of cervical cancer [7, 10].

In most LMIC, visual inspection with acetic acid (VIA) is the standard of care for cervical cancer screening, which requires trained health personnel to perform pelvic exams, and willingness by women to attend a clinic and undergo an invasive examination. This is a barrier to effective cervical cancer screening for many women in LMIC settings [11]. VIA brings additional challenges in WHIV who are more likely to have false positive examinations that lead to unnecessary and potentially harmful treatments. An innovative and feasible method of cervical cancer screening is emerging with the introduction of self-collection by vaginal swab for oncogenic HPV DNA testing. By avoiding the need for a pelvic examination this technology can significantly decrease the demand on trained healthcare providers with additional benefits of low cost and minimal infrastructure requirements [8, 12, 13]. With HPV DNA testing there is also the option to offer self-collection based screening where sample collection can be performed by women in their own homes, further reducing barriers. The Advances in Screening and Prevention in Reproductive Cancers (ASPIRE) project, based in Uganda, was developed to examine innovations in cancer prevention for women in LMIC [14–17]. Although other studies in sub-Saharan Africa have examined attitudes of WHIV to cervical cancer screening, none have specifically addressed self-collection of HPV as a screening modality in this population [18]. To further understand the attitudes of WHIV towards self-collected specimens for cervical cancer screening, we surveyed WHIV in Kisenyi, Uganda, to explore their intention to participate in self-collection based cervical cancer screening, and offered women self-collection based testing at the local HIV clinic. This information will guide programming to engage women with HIV in cervical cancer screening.

## Methods

### Design, setting and study population

The study design was a cross-sectional survey of knowledge, attitudes and behaviours towards self-collected sampling for cervical cancer screening in WHIV, who were then invited to participate in self-collection based screening. Interviews with WHIV and screening were conducted at a satellite HIV clinic of the Infectious Disease Institute (IDI) located at the Kisenyi Health Unit in an impoverished area of Kampala, Uganda. WHIV aged 30–69 years old attending a routine appointment for their HIV care were invited to participate by a member of the Friends Council, an HIV positive peer group between July and August 2013. An additional inclusion criterion was access to a mobile telephone. Women were excluded if they had previously had a hysterectomy, cervical cancer or they were unable to provide consent. Ethics approval for the study was obtained from the Research Ethics Board of the University of British Columbia (H09-02935-A013) and Makerere University (H13-02117).

### Survey & chart review

A structured questionnaire was created based on a previously validated survey using the Theory of Planned Behaviour exploring women’s intentions to provide a self-collected specimen for cervical cancer screening [14]. Items were reviewed with local and international experts in HIV and women’s health, revised and pilot tested prior to inclusion in the survey. The revised survey with additional HIV items was translated into the local language, Luganda, piloted in the community and then edited and revised based on feedback for clarity. Data was abstracted from each participant’s chart from the HIV clinic which included clinical information such as CD4 count, time since last appointment and blood work, World Health Organization (WHO) HIV disease stage [19], as well as type and duration of cART. In this clinical setting, women do not necessarily receive clinical blood work at each visit, but only if symptomatic or not on cART. Data from interviews and corresponding chart review were entered into an Access database created from survey questions and analyzed using SPSS (v14).

### Study procedures

In November 2013, local research assistants fluent both in English and Luganda were identified by the Ugandan ASPIRE research coordinator and trained in collaboration with the Canadian ASPIRE research team. In a private location that ensured patient confidentiality and allowed one-on-one interviews by the local research assistant, the survey participant’s primary language was used to explain the study, obtain informed consent, and complete the survey. After participants completed initial knowledge survey questions, the procedure of self-collecting a sample for HPV testing was explained by the local research assistant using a diagram of the swab, the genital tract, and cartoons of how to obtain self-collected sample. Surveys were completed by consenting study participants over a three-week period. Following completion of all survey and data recording, a workshop tailored from survey responses was held for study participants as an educational intervention about HIV and cervical cancer, HPV, and cervical cancer screening. This workshop also functioned to inform participants of relevant findings.

In February–June 2014, all participants were contacted by a nurse from the HIV clinic by phone and invited to make an appointment to participate in self-collection based HPV testing at the HIV clinic. Women who could not be reached by phone were approached at their next HIV follow up visit. At the time of their clinic visit, women were instructed on how to use the self-collected vaginal swab and asked to provide a sample in a private room at the clinic. Specimens were tested for high risk HPV (genotypes 16, 18, 31, 33, 35, 39, 45, 52, 56, 58, 59 and 66), *Neisseria gonorrhoeae* and *Chlamydia trachomatis* with real-time PCR. Women who tested HPV positive were contacted by phone with results and scheduled a colposcopy appointment for assessment at Mulago Hospital, a tertiary care center. Women who tested positive for *N. gonorrhoeae* and *C. trachomatis* were offered antibiotic treatment and counseling.

### Data analysis

Descriptive statistics for all survey questions, chart review data, and screening results were generated for all participants. Chi-square or Fisher’s exact test were used to compare factors of interest between HPV+ and HPV- women that participated in screening. Unadjusted odds ratios (OR) were calculated for all variables that reached significance of *p* > 0.05.

## Results

### Population demographics

In total 84 of 87 study participants completed the survey. Details for the demographic characteristics and chart review data of WHIV are captured in Table 1. WHIV in our study population were considered as engaged with health care with over 92.9% having attended for HIV care and monitoring in the past 6 months from chart review. Only 29.6% of women had been living with HIV for over 5 years, and 51.9% had a CD4 count over 350. The majority of women, 75.9%, had HIV WHO stage I or II disease [19] at the time of the study with 69% on an ART regimen.

**Table 1:** Demographics and chart review characteristics of WHIV

Variable		Total N = 87 N (%)
Age	30–35	36(43.4)
	36–40	29(34.9)
	41–45	7(8.4)
	46–50	5(6)
	51–55	2(2.4)
	56–60	4(4.8)
	61–69	0
	Missing	4
Marital status	Single	11(13.3)
	Married/Common law	29(34.9)
	Separated/Divorced	31(37.3)
	Widowed	12(14.5)
	Missing	4
Highest school level completed	No schooling/some primary	34(40.5)
	Primary/some secondary	46(54.8)
	Completed secondary school	2(2.4)
	Further studies (trade/college/university)	2(2.4)
	Missing	3
Work outside home	No	23(27.4)
	Yes	61(72.6)
	Missing	3
Accommodation	Rent	61(73.5)
	Own	22(26.5)
	No place to live	0
	Missing	4
Religion	Christian (various denominations)	67(79.8)
	Muslim	17(20.2)
	Missing	3
Ever had a pelvic examination	No	71(85.5)
	Yes	12(14.5)
	I don’t know	0
	Missing	4
Ever had sexual intercourse	Yes	84(100)
	Missing	3
Age at first sexual intercourse, median (IQR)		16(4)
Number of pregnancies, median (IQR)		4(3)
Years since HIV+ confirmed	<1 year	13(16.0)
	1–2 years	24(29.6)
	3–4 years	20(24.7)
	≥5 years	24(29.6)
	Missing	6
Mean Age diagnosed (SD)		33.96(6.54)
Most recent HIV appointment	<6 months	78(92.9)
	≥6 months	6(7.1)
	Missing	3
Most recent blood work	<6 months	39(44.8)
	≥6 months	42(51.9)
	Missing	3
Recent CD4 Count (Cells/mm^3^)	<350	39(48.1)
	≥350	42(51.9)
	Missing	3
WHO Stage	I	31(37.3)
	II	32(38.6)
	III	12(14.5)
	IV	8(9.6)
	Missing	4
Mean duration of ARV therapy in months (SD)		40.0(34.77)
On ARV	Yes	58(69.0)
	No	26(31.0)
	Missing	3

### Knowledge & intention to be screened

The survey responses for the knowledge, attitudes and behaviours among WHIV in this study are captured in Table 2. Less than 20% of WHIV reported having received education about cervical cancer screening, and over one third stated they never had been offered or advised to be screened. Most women had never heard of HPV (96.4%), while more than 60% of WHIV knew they were at increased risk for cervical cancer due to their HIV and were aware they needed to be screened. Of those that were aware of HPV, WHIV often knew that HPV risk was linked to sexual intercourse but unaware that HPV causes cervical cancer. Only 25% of WHIV reported condom use as their primary method of contraception, and the pill was the most popular method of contraception (27.4%), and 23.8% reported no contraception use.

**Table 2:** Knowledge, attitudes and behaviours among WHIV

Variable		Total N = 87 N(%)
Do you know someone who has had cervical cancer?	No	48(57.1)
	Yes	9(10.7)
	Don’t know	27(32.1)
	Missing	3
What do you think causes cervical cancer? *(open ended question, grouped by response)*	Sexual intercourse	30(35.7)
	Birth control	17(20.2)
	Poor hygiene	3(3.6)
	Other (ie cigarettes)	7(8.3)
	I don’t know	31(36.9)
	No response	27(32.1)
	Missing	3
Have you heard of Human Papillomavirus (HPV)?	No	81(96.4)
	Yes	3(3.6)
	Missing	3
Cervical cancer is caused by HPV	No	0
	Yes	23(27.7)
	I don’t know	60(72.3)
	Missing	4
What types of birth control have you and your partner used?	Condoms	21(25.0)
	Pill	23(27.4)
	Female Condom	0
	Do not use	20(23.8)
	Not sexually active	2(2.4)
	Other (injection, norplant, IUD, etc.)	31(36.9)
	Missing	3
During the last month, how frequently have you used condoms?	Never	53(63.1)
	Sometimes	16(19.0)
	Usually	12(14.3)
	Always	2(2.4)
	No having sex	1(1.2)
	Missing	3
Being tested for HPV is a way to screen for cervical cancer	No	3(3.6)
	Yes	37(44.6)
	I don’t know	43(51.8)
	Missing	4
Women with HIV are at increased risk for cervical cancer	No	0
	Yes	51(61.4)
	I don’t know	32(38.6)
	Missing	4
Screening for cervical cancer in women with HIV can prevent developing cancer of the cervix	No	3(3.6)
	Yes	52(61.9)
	I don’t know	29(34.5)
	Missing	3
Ever receive education about cervical cancer?	No	66(80.5)
	Yes	15(18.3)
	Don’t know	1(1.2)
	Missing	5
Cervical cancer screening ever advised	No	30(35.7)
	Yes	54(64.3)
	Missing	3
Reason no pelvic exam *(open ended question, grouped by response)*	Embarrassed about a pelvic exam	2(2.7)
	Thought it would be painful	5(6.8)
	Did not have time	0
	Other	2(2.7)
	Not offered a pelvic exam	64(87.7)
	Missing	14
Preferred place to self-collect	Home	33(39.3)
	Kisenyi Health Center	33(39.3)
	Both	18(21.4)
	Missing	4
I would be embarrassed to collect a sample at home	No	79(95.2)
	Yes	0
	Not sure	4(4.8)
	Missing	4
I am worried that I would not collect the sample properly	No	70(84.3)
	Yes	2(2.4)
	Not sure	11(13.3)
	Missing	4
I am afraid that HPV testing will show that I have cervical cancer	No	61(72.6)
	Yes	5(6.0)
	Not sure	18(21.4)
	Missing	4
I am afraid HPV testing will make other people think I have cervical cancer	No	82(97.6)
	Yes	0
	Not sure	2(2.4)
	Missing	3
I am worried self-collecting a sample will be painful	No	69(82.1)
	Yes	2(2.4)
	Not sure	13(15.5)
	Missing	3
I would be willing to have an outreach worker drop off a swab	No	22(26.5)
	Yes	58(69.9)
	Not sure	3(3.6)
	Missing	3
I would need my husband/partner’s approval to collect the sample	No	81(96.4)
	Yes	3(3.6)
	Missing	3
My religion/spiritual belief would affect my decision to be screened	No	84(100)
	Yes	0
	Not sure	0
	Missing	3
I do not think it is necessary to be screened for cervical cancer	No	0
	Yes	83(98.8)
	Not sure	1(1.2)
	Missing	3
Would you be willing to go to Kisenyi Health Center if the self-collected sample was found abnormal	No	1(1.2)
	Yes	83(98.8)
	Not sure	0
	Missing	3
I feel I am at risk for having HPV	No	40(47.6)
	Yes	7(8.3)
	Not sure	37(44.0)
	Missing	3

Overall 98.8% of WHIV intended to provide HPV self-collected specimens for cervical cancer screening. Only one woman indicated that she would not be willing to self-collect, and this was because she was pregnant at the time of the study. Despite the high acceptability of self-collection among WHIV, the majority of women did not think it was necessary to be screened for cervical cancer (98.8%). Similarly, perceived risk of HPV was low (8.3%), while 44% of WHIV were unsure if they were at risk. The vast majority of women (95.2%) did not see embarrassment or concerns of how to collect the specimen properly as barriers to screening; nor were they concerned others would perceive them to have cervical cancer if they were tested for HPV (97.6%), and 69.9% found it acceptable for an outreach worker drop off a swab at their home.

### Results from HPV testing

Of the 87 WHIV in the study, 40 (46.0%) were able to attend the clinic to provide a self-collected sample for high risk HPV testing. A total of 18/40 (45.0%) tested positive for one of the 12 high risk HPV genotypes and of those 6 (15.0%) women were positive for HPV strains 16 or 18. Among HPV positive women, 5/18 attended follow up colposcopy assessment, one of whom was positive for HPV 16/18, and all women were negative for cervical dysplasia. There were no cases of *N. gonorrhoeae* or *C. trachomatis* in the study population. Factors associated with HPV positivity are included in Table 3. WHIV who reported use of oral contraceptives were more likely to be HPV positive (OR = 6.65, 95% CI: 1.16, 38.19; *p* = 0.03) and WHIV who have had blood work within the past 6 months were more likely to be HPV positive (OR = 0.16, 95% CI: 0.03, 0.74; *p* = 0.02) (Table 4).

**Table 3:** Demographic/behavioural risk factor comparison between HPV+ and HPV- women

Variable	Variable level	Total *N* = (*N* = 40)	HPV results		
			HPV− (*N* = 22)	HPV+ (*N* = 18)	
			*N*(%)	*N*(%)	*p*-value
Age	Mean(SD)	39.05(6.28)	39.15(6.00)	38.94(6.78)	0.92
Marital status	Single	6(15.8)	4(19.0)	2(11.8)	0.59
	Married/Common law	13(34.2)	7(33.3)	6(35.3)	
	Separated/Divorced	14(36.8)	6(28.6)	8(47.1)	
	Widowed	5(13.2)	4(19.0)	1(5.9)	
Highest school level completed	No schooling/some primary	13(34.2)	7(33.3)	6(35.3)	1.00
	Primary/some secondary	24(63.2)	13(61.9)	11(64.7)	
	Completed secondary	1(2.6)	1(4.8)	0	
	Further studies (trade, college, university)	0	0	0	
How long to walk to nearest health centre?	Less than 10 min	7(18.4)	5(23.8)	2(11.8)	0.47
	Less than 30 min	13(34.2)	8(38.1)	5(29.4)	
	More than 30 min	18(47.4)	8(38.1)	10(58.9)	
Age at first sexual intercourse	Mean(SD)	16.83(2.83)	17.05(2.67)	16.53(3.11)	0.60
Type of contraception currently used	Condoms	9(23.7)	5(23.8)	4(23.5)	1.00
	Oral contraceptive	9(23.7)	2(9.5)	7(41.2)	0.05
	Do not use birth control	11(28.9)	5(23.8)	6(35.3)	0.68
	Not sexually active	0	0	0	
	Other (injection, norplant, IUD)	12(31.6)	8(38.1)	4(23.5)	0.49
Number of male partners in past week	None	16(42.1)	9(42.9)	7(41.2)	1.00
	One or more	22(57.9)	12(57.1)	10(58.8)	
Years since HIV+ confirmed	<1 year	4(10.8)	1(5.0)	3(17.6)	0.46
	1–2 years	12(32.4)	6(30.0)	6(35.3)	
	3–4 years	8(21.6)	4(20.0)	4(23.5)	
	≥5 years	13(35.1)	9(45.0)	4(23.5)	
Age diagnosed	Mean(SD)	34.61(6.39)	33.74(5.61)	35.59(7.21)	0.39
Most recent HIV appointment	<6 months	36(94.7)	20(95.2)	16(94.1)	1.00
	≥6 months	2(5.3)	1(4.8)	1(5.9)	
Most recent blood work	<6 months	12(32.4)	3(15.0)	9(52.9)	0.03
	≥6 months	25(67.6)	17(85.0)	8(47.1)	
Recent CD4 Count (Cells/mm^3^)	<350	9(24.3)	4(20.0)	5(29.4)	0.78
	≥350	28(75.7)	16(80.0)	12(70.6)	
WHO Stage	I	9(23.7)	5(23.8)	4(23.5)	0.75
	II	21(55.3)	10(14.6)	11(64.7)	
	III	4(10.5)	3(14.3)	1(5.9)	
	IV	4(10.5)	3(14.3)	1(5.9)	
On ARV	No	12(31.6)	4(19.0)	8(47.1)	0.13
	Yes	26(68.4)	17(81.0)	9(52.9)	
Medication currently taking	Well	1(2.6)	1(4.8)	0	1.00
	CBV	11(28.9)	7(33.3)	4(23.5)	0.72
	NVP	15(39.5)	12(57.1)	3(17.6)	0.02
	TDF	6(15.8)	4(19.0)	2(11.8)	0.67
	3tc	14(36.8)	9(42.9)	5(29.4)	0.61
	ATV	0	0	0	
	EFV	10(26.3)	4(19.0)	6(35.3)	0.29
	LPV	0	0	0	
	AZT	8(21.1)	5(23.8)	3(17.6)	0.71
	V	0	0	0

**Table 4:** Unadjusted odds ratio estimates for factors associated with HPV positivity

Variable	Variable Level	Unadjusted OR	95% CI	*p*-value
Currently use Oral contraceptive	No	Reference		
	Yes	6.65	1.16–38.19	0.03
Most recent blood work	<6 months	Reference		
	≥6 months	0.16	0.03–0.74	0.02

Among WHIV who participated in the study but did not attend screening, 2 of 47 could not be reached by phone, 5 of 47 indicated that they had screened for cervical cancer elsewhere, and 40 of 47 refused to attend the clinic. The main reasons for refusal were that distance to travel was too far, not having time to attend screening, or did not show up for the scheduled appointment.

## Discussion

### Knowledge of HPV, cervical cancer & intention to screen

Although our population in Kisenyi was highly engaged in HIV care, less than 20% had ever received any education around cervical cancer, 96% had never heard of HPV, and almost 99% did not feel it was necessary to be screened. These findings reflect a potential lack of cervical cancer training among HIV care providers, and competing health priorities in HIV positive populations. The low percentage of women in our study who had ever had a pelvic exam (14.5%) is further evidence of the potential impact that offering HPV self-collection as part of routine HIV care could have on WHIV to enhance the uptake of cervical cancer screening. Others have emphasized the need to integrate cervical cancer screening into routine HIV care for WHIV and have documented the impact of missed opportunities for education about cervical cancer by HIV care providers [20]. Despite this, data from South Africa, a country with significantly more health resources than Uganda, illustrates the positive impact of increased infrastructure on health education with over 85% of WHIV aware of cervical cancer screening [21].

### Self-collection based HPV testing for cervical cancer screening

In this group of WHIV engaged in care, there was a high prevalence of oncogenic HPV types (45.0%), a large proportion of which were HPV 16 or 18 (15%). This is much higher than other studies that ASPIRE has conducted in Kisenyi where HPV positivity rates among HIV negative women was only 28.9%, of which 5.3% were HPV 16 or 18 [22]. Our HIV positive population was more likely to live or work outside of Kisenyi, compared to past studies where self-collection was offered by community outreach workers at their homes [23, 24]. This suggests that a model for screening with self-collection for WHIV may be more appropriately based out of a health center, as these women are already engaged in care, thereby avoiding unnecessary travel. Unlike the present study where many women were asked to attend self-collection outside of their normal HIV appointment schedule; had screening been integrated with routine HIV care, uptake and follow-up would have undoubtedly been higher. Uptake of self-collection based screening in Kisenyi was 99% in a recent study [24], and a subgroup analysis demonstrated 95% uptake among WHIV [22]. Similarly, we believe that the distance to the tertiary care hospital where colposcopy is conducted, as well as the time required for the additional visit were barriers to follow up assessment. Competing priorities, lack of perceived risk, and cost are likely important factors that have made colposcopy follow up adherence a challenge in this, and other studies our team has conducted in this community [24].

In our population, women who were taking oral contraceptives were more likely to be HPV positive. This is consistent with other studies, although the literature around the role of oral contraceptive on HPV positivity is unclear and in need of further study [25, 26]. Women who had taken blood work within the last 6 months were also more likely to be HPV positive. Since blood work is usually only performed on women who are symptomatic, it is possible that HPV is more persistent in these women.

### Limitations and policy implications

This study was limited by its small sample size, which prevented us from conducting further statistical testing such as logistic regression. Another limitation was that a large proportion of our original survey population was unavailable to attend screening. Due to limitations on the study timeline, we were unable to offer screening during a routine HIV clinical visit to demonstrate an integrated approach. Despite this, we feel our results demonstrate the feasibility of a self-collection based approach to screening, while underscoring the challenges of offering interventions in a siloed manner.

Despite alarmingly low levels of knowledge of HPV, global expansion of the HPV vaccine in countries including Uganda offers the opportunity for community health workers to engage and educate girls, women and their families on cervical cancer prevention. Self-collection for HPV screening has been demonstrated as a highly acceptable and effective method of cervical cancer screening, particularly in susceptible populations such as WHIV [14, 15]. This highly scalable model can be adapted and implemented in various ways to improve access using minimal infrastructure. In community-based models for HPV self-collection, community outreach workers can be used to offer women screening at their homes or places of work. There is also huge potential for this type of screening to be offered in an integrated manner, where screening can be combined with other health services such as maternal/child or reproductive health services. Given the higher rates of cervical cancer among WHIV, screening should without question be integrated with routine HIV appointments. HIV clinics are often already equipped with the necessary human resources and infrastructure needed to implement self-collection based screening and follow up, including a laboratory where HPV testing can be performed. Of note, colposcopy and early curative treatment would need to be more readily accessible for effective scale up of a comprehensive cervical cancer screening program.

## Conclusions

To not integrate prevention of cervical cancer in HIV care would do a great disservice to the accomplishments that have been made in the management of HIV. The overwhelming and growing need for accessible and effective screening in WHIV at risk for cervical cancer should compel us to look at feasible methods on a large scale. Our study of WHIV in this LMIC setting demonstrates the potential for HPV self-collection to be integrated into routine HIV care at a community level.

## Abbreviations

ASPIRE: The advances in screening and prevention in reproductive cancers project
cART: Combination antiretroviral treatment
CD4: Cluster of differentiation 4
HIV: Human immunodeficiency virus
HPV: Human papillomavirus
IDI: Infectious Disease Institute
LMIC: Low and middle income country
OR: Odds ratio
WHIV: Women living with HIV
WHO: World Health Organization
